# Identification of *An7* as a positive awn regulator from two wild rice species

**DOI:** 10.1270/jsbbs.23052

**Published:** 2024-06-19

**Authors:** Miya Mizutani, Riri Murase, Shin-ichiro Aoki, Yutaka Sato, Yoshiyuki Yamagata, Hideshi Yasui, Atsushi Yoshimura, Motoyuki Ashikari, Kanako Bessho-Uehara

**Affiliations:** 1 Bioscience and Biotechnology Center, Nagoya University, Nagoya, Aichi 464-8601, Japan; 2 Graduate School of Bioagricultural Sciences, Nagoya University, Nagoya, Aichi 464-8601, Japan; 3 National Institute of Genetics, Mishima, Shizuoka 411-8540, Japan; 4 Plant Breeding Laboratory, Faculty of Agriculture, Kyushu University, 744 Motooka, Nishi-ku, Fukuoka 819-0395, Japan; 5 Graduate School of Life Sciences, Tohoku University, Sendai, Miyagi 980-8578, Japan

**Keywords:** *An7*, awn, *Oryza melidionalis*, *Oryza glumaepatula*, brassinosteroid

## Abstract

The awn is a bristle-like appendage that protrudes from the seed tip and plays a critical role in preventing feed damage and spreading habitats in many grass species, including rice. While all wild species in the *Oryza* genus have awns, this trait has been eliminated in domesticated species due to its obstructive nature to agricultural processes. To date, several genes involved in awn development have been identified in wild rice, *Oryza rufipogon* and *Oryza barthii* which are ancestral species of cultivated rice in Asia and Africa, respectively. However, the responsible genes for awn development have not been identified in other wild rice species even though multiple QTLs have been reported previously. In this study, we identified *An7* gene responsible for awn development in two wild rice species, *Oryza glumaepatula* and *Oryza meridionalis*. *An7* encodes a cytochrome P450 enzyme and is homologous to *D2/CYP90D2*, a known brassinosteroid biosynthesis enzyme in rice. The identification of *An7* provides insight into a distinct molecular mechanism underlying awn development that occurs in geographically separated environments.

## Introduction

Wild rice retains a variety of morphological, physiological, and ecological characteristics that make it an important genetic resource. For example, *Oryza longistaminata*, a wild African rice species, has strong bacterial blast resistance and *Xa21* gene contributes to this ability (Song *et al.* 1995). The *O. longistaminata* allele of *Xa21* has been introduced into a cultivated rice variety to accomplish the high blast resistance (Nguyen *et al.* 2018). On the contrary, traits considered to be detrimental in agriculture have been selected and eliminated during the rice domestication. Awn is the one example of eliminated traits in rice. Awn is a bristle-like appendage at the tip of lemma and helps to avoid feeding damage and seed dispersal. All wild rice species have awns, however, cultivated species have been lost this trait. Several genes responsible for awn development have been selected during rice domestication. In Asia, loss of function of three genes were identified as the main causes for the loss of awn during rice domestication. *An-1* (Luo *et al.* 2013), which is also detected as *Regulator of Awn Elongation 1* (*RAE1*) (Furuta *et al.* 2015) encoding bHLH transcription factor, *LABA1* which encodes cytokinin activating enzyme (Hua *et al.* 2015), and *RAE2* (Bessho-Uehara *et al.* 2016), which is also annotated as *GAD1* (Jin *et al.* 2016) encoding OsEPFL1 signal peptide were selected during Asian rice domestication. On the other hand, in Africa, single gene named *RAE3* which encodes an E3 ubiquitin ligase was identified as the main responsible gene for the loss of awn (Bessho-Uehara *et al.* 2023). Among these genes, *An-1/RAE1* and *RAE2/GAD1* genes are commonly conserved in most wild rice species according to comparative analysis of several chromosome segment substitution lines (CSSLs) (Bessho-Uehara *et al.* 2021). Through the genetic analysis conducted by Ikemoto *et al.*, it was revealed that *An-1/RAE1* and *RAE2/GAD1* complement awn phenotype around 70% of *O. rufipogon* in the *O. sativa* genetic background (Ikemoto *et al.* 2017). However, interestingly, the backcross line carrying *An-1/RAE1* and *RAE2/GAD1* genes from *O. sativa* (dys-functional alleles) in *O. rufipogon* background, it was observed that the awn length remained long, approximately 70% of that of *O. rufipogon* (Ikemoto *et al.* 2017). These findings indicate *An-1/RAE1* and *RAE2/GAD1* possess large effects on awn elongation, *vice versa*, multiple other genes with small effects that work in concert in awn elongation.

More than ten QTLs related to rice awn have been identified (Cai and Morishima 2002, Fawcett *et al.* 2013), but only some responsible genes have been identified and the molecular mechanism elucidated (Bessho-Uehara *et al.* 2016, 2023, Furuta *et al.* 2015, Hua *et al.* 2015, Jin *et al.* 2016, Luo *et al.* 2013, Toriba and Hirano 2014). To understand the comprehensive molecular mechanism of awn development, we need to identify more responsible genes of awnness in rice.

Previous studies on genes associated with awn development have mainly relied on analyses using domesticated rice and its wild ancestor species. However, since there are 22 wild rice species, all of which possess long awns, it was hypothesized that new genes involved in awn elongation could be discovered. Previous researches using inbred lines between *O. sativa* and either *O. meridionalis* or *O. glumaepatula* suggested the involvement of genes named *An7* and *An8* in awn elongation (Kurakazu *et al.* 2001, Matsushita *et al.* 2003a, 2003b). The gene *An8* on chromosome 4 is presumed to be *An-1/RAE1* according to its locus position. However, *An7* on chromosome 5 has not been identified. In this study, we employed inbred lines to narrow down the *An7* gene to shed light on its contribution to the complex genetic control of awn development.

## Materials and Methods

### Plant materials and growth conditions

*O. sativa* ssp. *japonica* ‘Taichung 65 (T65)’, *O. glumaepatula acc.* IRGC105668, *O. meridionalis acc.* W1625, and two lines retrieved from chromosome segment substitution lines (CSSLs) were employed for evaluation of awn phenotype. Two CSSL lines, Glu-IL115 and Mer-IL116 were used to map *An7* gene that controls awn development. Glu-IL and Mer-IL are CSSLs that carry chromosome segments from *O. glumaepatula* and *O. meridionalis*, respectively, in T65 genetic background (Yoshimura *et al.* 2010). Seeds of all lines are provided from Kyushu University. Glu-IL115 harbors chromosome 1 (25,071,234-31,948,582 bp) and chromosome 5 (5,284,924-21,426,063 bp) segments substitutions from the donor parent, *O. glumaepatula acc.* IRGC105668. Mer-IL116 harbors a chromosome 5 (2,881,341-16,811,499 bp) segment substitution from the donor parent, *O. meridionalis acc.* W1625. These plant materials were grown either in the greenhouse at Nagoya University, Furo-cho, Nagoya, Aichi or in the research field of Nagoya University, Togo, Aichi, Japan with conventional agricultural calendar under natural conditions. Seedlings were first grown in the greenhouse for about 30 days and then transplanted in the greenhouse or the field. The transgenic plants were grown in isolated greenhouses under long-day conditions until the 10-leaf stage, and then transferred to short-day conditions until flowering.

### Evaluation of morphological phenotypes

Panicles of the parental lines, CSSLs and transgenic lines were harvested after seed maturation for evaluation. Five to ten plants were randomly selected for phenotypic evaluation. The length of the awn was taken as the distance from the tip of lemma to the end of awn. The length of the awn was measured by a ruler on the first spikelet located on the primary branch of each panicle. The average values of at least five panicles were calculated for each plant. Five hundred grains were used for measuring grain length and grain width by ImageJ (version 1.51n). Fully filled grains were used to measure 1,000-grain weight, and five replicates were performed. The statistical phenotypic data was analyzed using student t-test for significant difference.

### DNA extraction and genotyping of the *An7* mapping population using SSR markers

For linkage analysis of *An7*, we used 960 BC_4_F_3_ plants produced by inbreeding BC_4_F_2_ plants of Glu-IL115 and Mer-IL116 whose chromosome 5 substitution regions are heterozygous, respectively. Segregation ratio of BC_4_F_3_ was evaluated by chi-square test to calculate the deviation from Mendelian ratios. Genomic DNA from the mapping population was extracted following the TPS method (Hattori *et al.* 2009). The polymerase chain reaction (PCR) analysis was performed in a 10 μL reaction mixture containing 50 mM KCl, 10 mM Tris-HCl (pH 9.0), 1.5 mM MgCl_2_, 200 μM of each dNTP, 0.2 μM of each primer, 0.75 U of Taq polymerase (Takara, Otsu, Japan), and approximately 10 ng of template DNA in a thermal cycler (cat. 9700N8050200, Applied Biosystems, Foster City, CA, USA). The PCR program was 95°C for 5 min for initial denaturation, followed by 35 cycles at 95°C for 30 s, 55°C for 30 s and 72°C for 30 s, with no final extension. PCR products were run in 3% agarose gels in 0.5× Tris-borate-EDTA (TBE) buffer containing ethidium bromide. Primer sequence information of single sequence repeat (SSR) markers is shown in Table 1.

### Plasmid construction and generation of transgenic plants

For complementation test, the *An7* candidate genes, *LOC_Os05g11120*, *LOC_Os05g11130*, and *LOC_Os05g11160*, were amplified by PCR using Glu-IL115 genomic DNA as templates and *LOC_Os05g11140* was amplified by PCR using Glu-IL115 complementary DNA as a template. Primers were designed in a region about 3-kb upstream and 2-kb downstream to contain the promoter and terminator for *LOC_Os05g11120*, *LOC_Os05g11130*, and *LOC_Os05g11160*. The amplified fragment was introduced into the pCAMBIA1380 vector and written as own promoter::*An7*-candidate gene ^Glu-IL115^/pCAMBIA1380. Insertion of the fragments was confirmed by Sanger sequencing using ABI3730 (Thermo Fisher Scientific). For LOC_Os05g11140, its amplified fragment was integrated into the ΩpCAMBIA1380 vector and written as Ubiquitin::*An7*-candidate gene ^Glu-IL115^/ΩpCAMBIA1380. These constructs were introduced into callus of T65, which is an awnless line, using *Agrobacterium tumefaciens* (EHA105)-mediated transformation method (Hiei *et al.* 1994). The line transformed empty vector of pCAMBIA1380 is used as a vector control. For producing *RAE3* mutation in Glu-IL115, a construct in pMgPoef4_129-2A-GFP vector backbone with gRNA targets designed on three locations of *RAE3* was generated according to the previous manuscript (Mikami *et al.* 2015). Using *A. tumefaciens* (EHA105)-mediated transformation, the construct was introduced into callus of Glu-IL115, which is an awnled line. pMgPoef4_129-2A-GFP, a vector without guide RNA sequences, was used as a vector control. Primers used to make constructs are listed in Table 1.

### Gene annotation, sequencing, and amino acid alignment

The reference genome of *O. glumaepatula* and *O. meridionalis* was retrieved as GCA_000576495.1 (*O. glumaepatula acc.* GEN1233_2) and GCA_000338895.2 (*O. meridionalis acc.* W2112) from Gramene (https://www.gramene.org/). The short reads of IRGC105668 (*O. glumaepatula*) and W1625 (*O. meridionalis*) were retrieved from DRR057991 and DRR058014 respectively, registered in NCBI SRA database. MEGANTE (Numa and Itoh 2014) was used for gene prediction in *O. glumaepatula* and *O. meridionalis*. The Rice Genome Annotation Project (http://rice.uga.edu/) was used for *O. sativa* gene annotation. Four candidate genes were amplified with Prime Star GXL (Takara) using T65 and Glu-IL115 genomic DNA as templates. Then, PCR products are sequenced with BigDye Terminator (Thermo Fisher Scientific) and ABI3730 (Thermo Fisher Scientific). Based on the CDS sequence we acquired, amino acid sequence was predicted comparing with the CDS sequence of *LOC_Os05g11130*. The multiple sequence alignment of An7 protein sequences in *O. sativa*, *O. glumaepatula* and *O. meridionalis* were performed by MUSCLE program using Geneious software (version 9.0) with default settings.

### Visualization of local genome synteny around the candidate region of *An7*

To visualize differences in genome structure around the candidate region of *An7*, the genome sequences of *O. sativa*, *O. glumaepatura* and *O. meridionalis* were compared. For the candidate region of *An7*, a specific segment (positions 6,254,283 to 6,305,695 bp) on chromosome 5 of *O. sativa* was retrieved from Nipponbare genome sequence IRGSP-1.0, distributed via the RAP-DB (https://rapdb.dna.affrc.go.jp/). The corresponding genomic regions in *O. glumaepatula* and *O. meridionalis*, aligning with the *An7* candidate region in *O. sativa*, were identified based on BLAST hits of the first and last 100 bp of the *O. sativa* subset sequence. The reference sequences of *O. glumaepatura* and *O. meridionalis* were obtained as described above. The blastn program of bl2seq was performed using GenomeMatcher ver. 2.3 (Ohtsubo *et al.* 2008) with default parameter. The local genomic sequence corresponding to the candidate region of *An7* was visualized using GenomeMatcher, with adjustments made to exclude lines that connected sequences showing relatively low similarity (less than 70%).

### Constructing phylogenetic tree

Amino acid sequences of *An7* homologues from various plant species were identified through reciprocal best-match BLAST searches using Phytozome v13 database (https://phytozome-next.jgi.doe.gov). Accession numbers and locus identifications were derived from Phytozome v13. Amino acid sequences were aligned using the MUSCLE program implemented in Geneious software version 6.1.6 (Biomatters; http://www.geneious.com/) with default parameters. The phylogenetic tree was constructed by the maximum likelihood method using PhyML 3.0 (Guindon *et al.* 2010). Branch nodes were labeled with a cutoff at 500, using bootstrap values calculated from 1000 replications.

### RNA isolation and quantitative Real-Time (qRT) PCR

For qRT PCR analysis of *An7*, young panicles (length is less than 5 mm) and spikelets which are in the stage of before heading (panicle length is around 10 cm) of T65, Glu-IL115 and Mer-IL116 were used. Total RNA was extracted by RNeasy Plant Mini Kit (QIAGEN), then first-strand cDNA synthesis was performed using the Omniscript RT Kit (QIAGEN). StepOneTM Real-Time PCR system (Applied Biosystems) was used to analyze the expression levels of *An7*. Relative expression levels of the target genes were normalized to the levels of endogenous ubiquitin transcripts (*LOC_Os02g06640*). The experiment was repeated three times and the Comparative CT method (ΔΔCT Method) was used to calculate the relative expression levels of the target genes. The error bars display the calculated maximum (RQMax) and minimum (RQMin) expression levels that represent standard error of the mean expression level (RQ value).

## Results

### Phenotypic evaluation of CSSLs and their parents

**Fig. 1.**
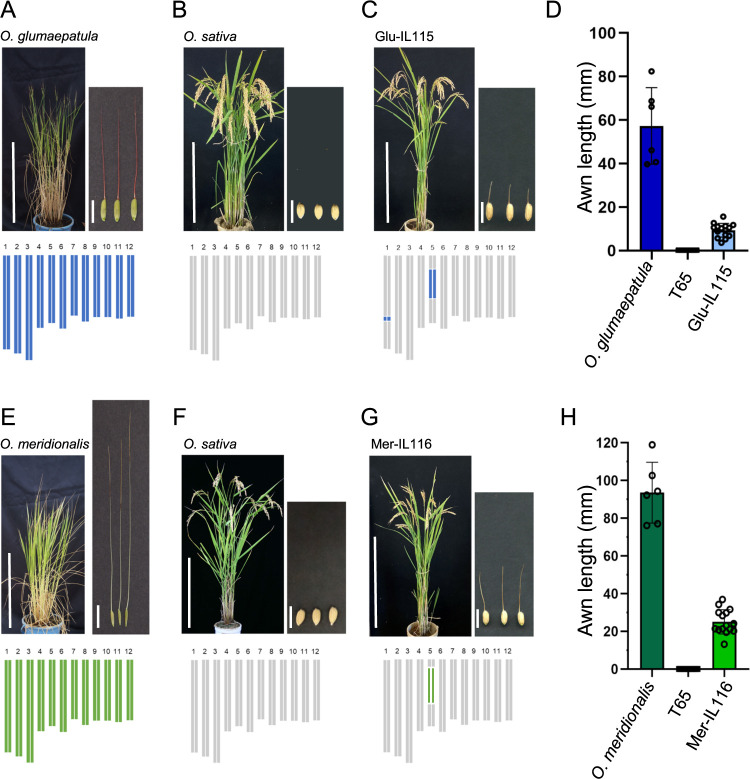
Plant materials used in this study and their awn phenotype. (A–D) Phenotypic comparison of awns in South American wild rice, *O. glumaepatula acc.* IRGC105668 (A), *O. sativa* ssp. *japonica* ‘T65’ (B) and Glu-IL115 (C). Awn length of *O. glumaepatula*, T65, and Glu-IL115 (D). (E–H) Phenotypic comparison of awns in Oceanian wild rice, *O. meridionalis acc.* W1625 (E), T65 (F) and Mer-IL116 (G). Awn length of *O. glumaepatula*, T65, and Mer-IL116 (H). Twelve rectangles below each photo represent the rice chromosomes with colors indicating species; blue, *O. glumaepatula*; green, *O. meridionalis*; gray, *O. sativa*. Left panels display whole plants (bars = 50 cm) and right panels show seeds and awns (bars = 1 cm). Awn length data were measured from 6 to 15 seeds, with error bars representing mean ± SD.

Previous studies using the inbred lines between *O. sativa* and *O. glumaepatura* or *O. meridionalis* had shown that *An7* is located on short arm of chromosome 5 of the respective wild rice species (Kurakazu *et al.* 2001, Matsushita *et al.* 2003a, 2003b). It was estimated that the genes on chromosome 5 detected in these two species were identical but with different alleles according to the overlap of gene location. Prior to identification of *An7* in wild rice *O. glumaepatula* and *O. meridionalis*, awn phenotype of CSSLs and their parents was evaluated. *O. glumaepatula* acc. IRGC105668 formed a 56.9 mm long awn on average, while *O. sativa* ssp. *japonica* ‘T65’ formed no awn (Fig. 1A, 1B, 1D). The CSSL Glu-IL115, which had a substitution of a portion of its chromosomes 1 and 5 with those of *O. glumaepatula*, formed an awn of approximately 10 mm in length (Fig. 1C, 1D). However, other CSSLs, namely Glu-IL103 and Glu-IL107, which harbored a segment substitution in the corresponding region on chromosome 1 from *O. glumaepatula*, did not form awns. This led us to conclude that the segment on chromosome 5 from *O. glumaepatula* was responsible for awn elongation in Glu-IL115. W1625 which is an accession of *O. meridionalis* formed awns that reached 80.4 mm long (Fig. 1E, 1F, 1H), while the CSSL, Mer-IL116, which had a substitution of a segment of its chromosome 5 with that of *O. meridionalis*, formed an awn of approximately 25.2 mm in length (Fig. 1G, 1H). These results indicated that both alleles of *An7* have positive effect on awn development.

### Mapping of *An7* as responsible gene for awn elongation in *O. glumaepatula* and *O. meridionalis*

To investigate the inheritance pattern of *An7*, we observed an awn phenotype in a randomly selected population of approximately 100 individuals from the BC_4_F_3_ generation. Based on the results of Glu-IL115 and Mer-IL116, the segregation of awned versus awnless individuals followed a ratio of 72:24 and 73:21, respectively. These ratios showed a good fit to the expected 3:1 ratio, as indicated by the chi-square test, suggesting a dominant inheritance pattern for *An7*.

**Fig. 2.**
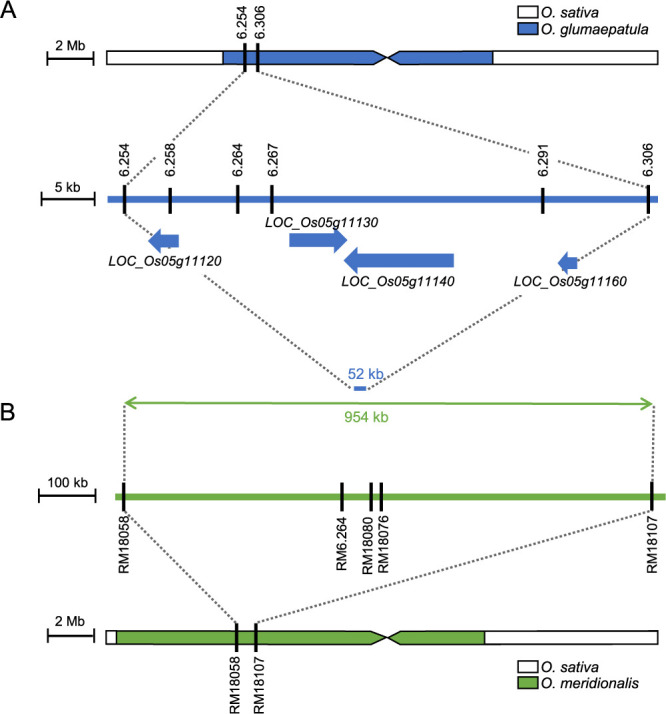
Mapping of *An7.* (A, B) Mapping results of *An7* using progenitor of Glu-IL115 (A) and Mer-IL116 (B). White bars represent chromosome 5 of T65 (*O. sativa*). Blue and green bar represent chromosome segment of *O. glumaepatula* and *O. meridionalis*, respectively. Black vertical lines denote DNA marker locations, and the numbers corresponding to marker names. Blue arrows indicate four genes annotated within 52 kb of candidate region in Glu-IL115 by MEGANTE.

To map *An7* in *O. glumaepatula* and *O. meridionalis*, we used 960 lines from the BC_4_F_3_ populations of Glu-IL115 and Mer-IL116, respectively. Through genotyping and phenotyping, we identified 62 recombinant lines with awn and 20 recombinant lines without awn in the Glu-IL115 progenies, which narrowed down the candidate region of *An7* in *O. glumaepatula* to approximately 52 kb on the short arm of chromosome 5 (Fig. 2A). Similarly, in the Mer-IL116 progenies, we found 93 awned recombinant lines and 54 awnless recombinant lines, resulted in the candidate region of *An7* in *O. meridionalis* to approximately 954 kb on the short arm of chromosome 5 (Fig. 2B). These candidate regions overlapped, indicating that *An7* in *O. glumaepatula* and *O. meridionalis* may be identical. Within the 52 kb candidate region in Glu-IL115, there were 5 genes, *LOC_Os05g11120*, *LOC_Os05g11130*, *LOC_Os05g11140*, *LOC_Os05g11150*, and *LOC_Os05g111600*, located in Nipponbare (*O. sativa* ssp. *japonica*) genome in Rice Genome Annotation Project (http://rice.uga.edu/, searched on March 29, 2022). Among them, four genes were predicted within the 52 kb candidate region in *O. glumaepatula* by MEGANTE (https://megante.dna.affrc.go.jp/, searched on March 29, 2022). One of 5 genes annotated in *O. sativa*, *LOC_Os05g11150*, was not predicted in *O. glumaepatula*. Considering the result of awn phenotype segregation and the functional presence of *An7* in *O. glumaepatula*, *LOC_Os05g11150* was excluded as a candidate gene for *An7* in *O. glumaepatula*. Thus, we narrowed down the candidate genes for *An7* into four genes in *O. glumaepatula* (Fig. 2A).

**Fig. 3.**
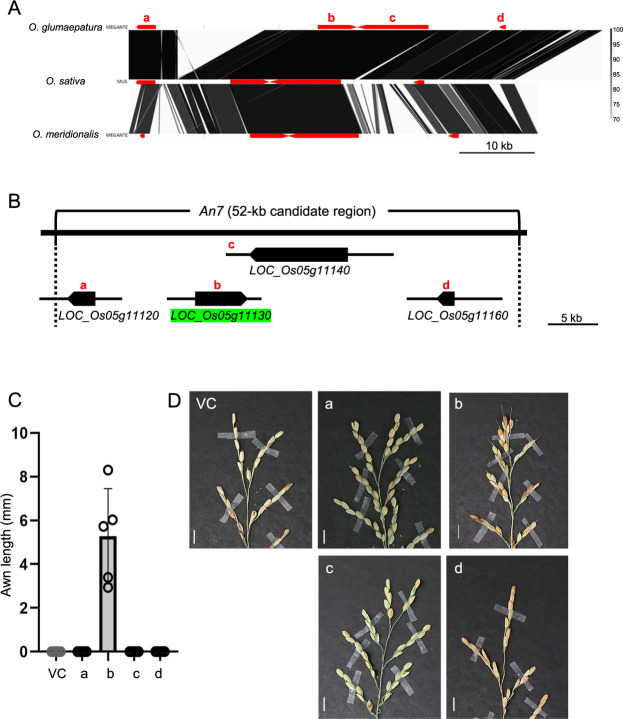
Complementation test of *An7* candidate genes. (A) Regional synteny around the *An7* candidate region on chromosome 5 compared among *O. glumaepatula*, *O. sativa*, and *O. meridionalis*. A color scale for percent identity shown on the right. Lower case letters, a to d, indicate genes; a, *LOC_Os05g11120*; b, *LOC_Os05g11130*; c, *LOC_Os05g11140*; d, *LOC_Os05g11160*. (B) Genomic regions utilized for the complementation test. Pentagons indicate gene body and black horizontal lines above pentagons reoresenting promoter and terminator regions of each gene. (C) Awn lengths of the transgenic lines used for complementation test. We used T65 as the recipient for the transgenes. Error bars represent mean ± SD. (D) Awn phenotypes of complementation test lines. VC, vector control; a to d indicate the genes described above. Scale bars = 10 mm.

By comparing the regional genomic synteny, we aligned the genomic sequence of 52-kb candidate region among *O. sativa*, *O. glumaepatura* and *O. meridionalis* (Fig. 3A). We found few SNPs between *O. glumaepatula* and *O. sativa* in the candidate region, in addition to a 10-kb deletion between *LOC_Os05g11120* and *LOC_Os05g11130*. On the contrary, there are many in-dels between *O. meridionalis* and *O. sativa* in this region. To identify the functional *An7* allele from Glu-IL115 that induces awn elongation in the genetic background of *O. sativa* ssp. *japonica* ‘T65’, a complementation test was conducted. Genomic fragments, including the promoter and terminator regions of each gene, were cloned from the genomic DNA of Glu-IL115 (Fig. 3B). Among the transgenic lines, only those carrying *LOC_Os05g11130*^Glu-IL115^ showed complementation of the awn phenotype with an average awn length of 5.8 mm (Fig. 3C, 3D). Based on these results, we concluded that *LOC_Os05g11130*^Glu-IL115^ is the responsible gene for *An7*.

### *An7* encodes cytochrome P450 enzyme and locates in the sister clade of *D2*

**Fig. 4.**
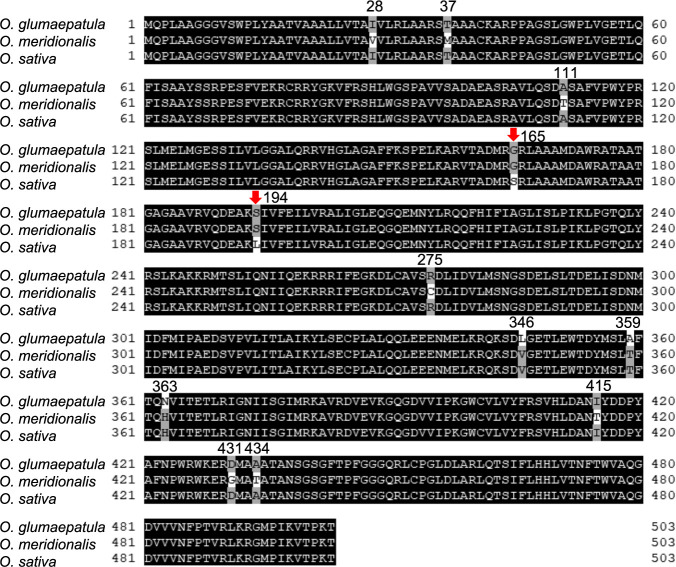
Comparative analysis of the protein sequence of *An7*. A comparison of the amino acid sequences of An7 between *O. sativa*, *O. glumaepatula* and *O. meridionalis*. The sequence positions are indicated by numbers representing amino acid locations in An7. Non-synonymous variations, as compared with wild rice and *O. sativa*, are marked with red arrows.

**Fig. 5.**
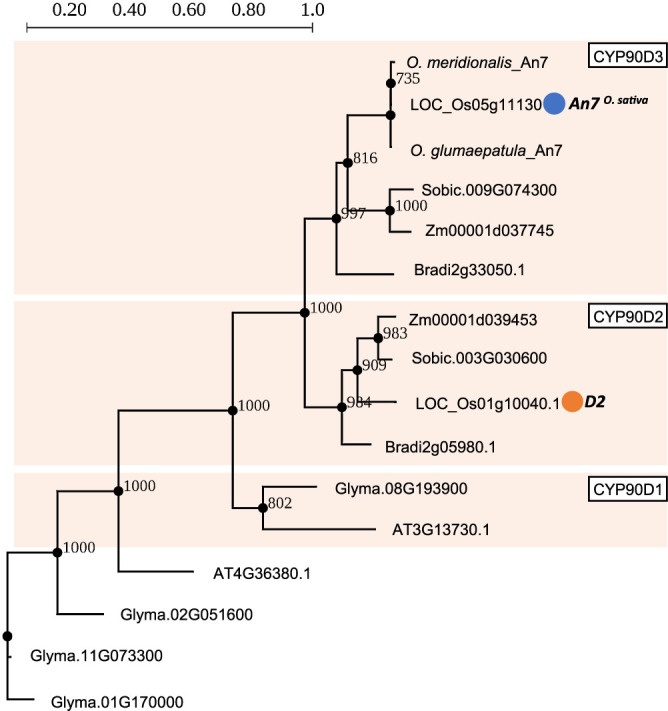
Phylogenetic tree of *An7* homologues across various plant species. Phylogenetic tree was generated using the *An7* homologues from several plant species, *A. thaliana* (AT), *O. sativa* (LOC_Os), *O. glumaepatula*, *O. meridionalis*, *Sorghum bicolor* (Sobic), *Zea mays* (Zm), *Brachypodium distachion* (Bradi), and *Glycine max* (Glyma). The tree was constructed using the maximum likelihood method with PhyML, with 1000 bootstrap trials. Bootstrap values at key nodes are displayed. Scale bar indicates number of amino acid changes per branch length. *An7* is marked with a blue circle, and *D2* which is previously reported as brassinosteroid biosynthesis enzyme is marked with an orange circle.

According to the sequence, *An7* encodes cytochrome P450 CYP90D3 gene. To elucidate the *An7* mutation for loss-of-function in *O. sativa*, we compared the amino acid sequence of An7 among *O. sativa*, *O. glumaepatula* and *O. meridionalis*. Two non-synonymous *O. sativa* specific mutations are present (Fig. 4). Considering that almost the entire portion of An7 encodes a cytochrome P450 domain (14L to 474F), these two mutations could cause a decrease in affinity for its substrate. According to the phylogenetic tree of *An7* and its homologues, *An7* is located in CYP90D3 clade (Fig. 5), which is next to CYP90D2 clade including *D2*, which is previously reported brassinosteroid (BR) biosynthesis enzyme in rice (Hong *et al.* 2003). These results suggested that *An7* carries cytochrome P450 domain and might be involved in BR biosynthesis.

### The functional alleles of *An7* are highly expressed in young panicle

**Fig. 6.**
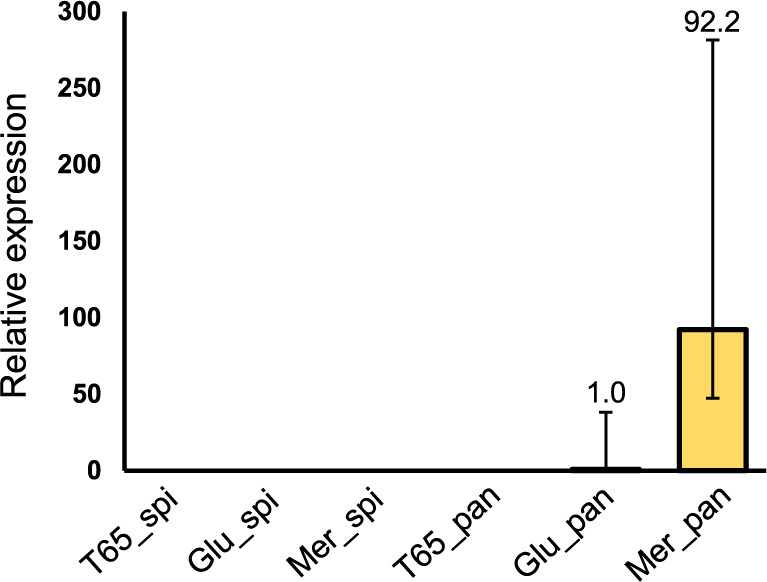
Expression pattern of *An7* in *O. sativa* and CSSLs. Relative expression levels of *An7* in spikelets and young panicle of T65 (*O. sativa* allele), Glu-IL115 (*O. glumaepatula* allele) and Mer-IL116 (*O. meridionalis* allele). *OsUBI* was used as an internal control. Spikelet and panicle samples are denoted as ‘spi’ and ‘pan’, respectively. Error bars represent the calculated maximum (RQMax) and minimum (RQMin) expression levels, indicating the standard error of the mean expression level (RQ value).

To examine the expression pattern of *An7*, we performed quantitative Real-Time (qRT) PCR analysis using *O. sativa* and CSSLs (Glu-IL115 carrying *O. glumaepatura* allele of *An7*, and Mer-IL116 carrying *O. meridionalis* allele of *An7*). A previous study reported that *CYP90D3* is expressed specifically in the root of *O. sativa* (Hong *et al.* 2003). We collected floral organs such as young panicle (length <5 mm) and spikelet (after heading) in order to examine the relationship between *An7* expression and awn development. No expression of *An7* in *O. sativa* was detected in young panicle whereas *An7* was expressed in both CSSLs. In addition, the relative expression level of *An7* in young panicle of Mer-IL116 was approximately 90 times higher than that of Glu-IL115 (Fig. 6). This result indicated that *An7* allele of *O. glumaepatula* is highly expressed in comparison to that of *O. sativa*, but is significantly lower than that of *O. meridionalis* in young panicles. There are no expressions in spikelets of all three lines. This result suggested that *An7* functions in young panicle to elongate awn in both *O. glumaepatura* and *O. meridionalis*, and this expression could be diminished in the later stage.

### *An7* functions cooperatively with *RAE3* for awn elongation in rice

**Fig. 7.**
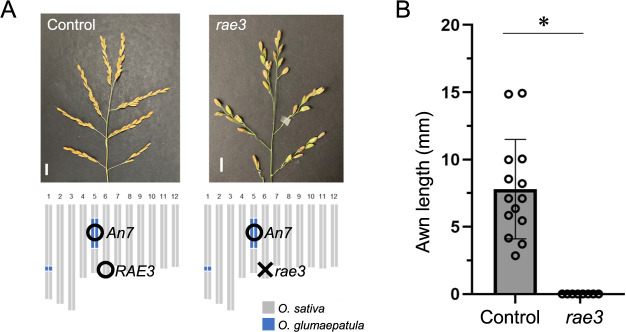
Knockout mutant of *RAE3* in the background of Glu-IL115 results in an awnless phenotype. (A) Awn phenotype and graphical genotype of the control (Glu-IL115, left) and *RAE3* CRISPR line (right, indicated as *rae3*). Scale bars represent 1 cm. (B) Awn length of the control and *RAE3* CRISPR line (indicated as *rae3*). Awn length were measured from 15 or more seeds, with error bars representing mean ± SD. An asterisk indicates P < 0.05 by student’s t-test.

The CSSL line, Glu-IL115 carries functional alleles of both *An7* and *RAE3*. Since *RAE3* encodes an E3 ubiquitin ligase and works cooperatively with *An-1/RAE1* and *RAE2/GAD1* for awn elongation (Bessho-Uehara *et al.* 2023), there were two hypotheses regarding the relationship between *An7* and *RAE3*. First, *An7* may work independently for awn elongation with *RAE3*. Second, *An7* may work cooperatively with *RAE3* for awn elongation. To understand the relationship between *An7* and *RAE3*, we generated a knockout line of *RAE3* in Glu-IL115 background using CRISPR/Cas9 system (Fig. 7A). The *RAE3* knockout line showed an awnless phenotype even *An7* is functional (Fig. 7B), indicating that *An7* works cooperatively with *RAE3* for awn elongation in rice.

### *An7* has the potential to affect grain phenotype pleiotropically

**Fig. 8.**
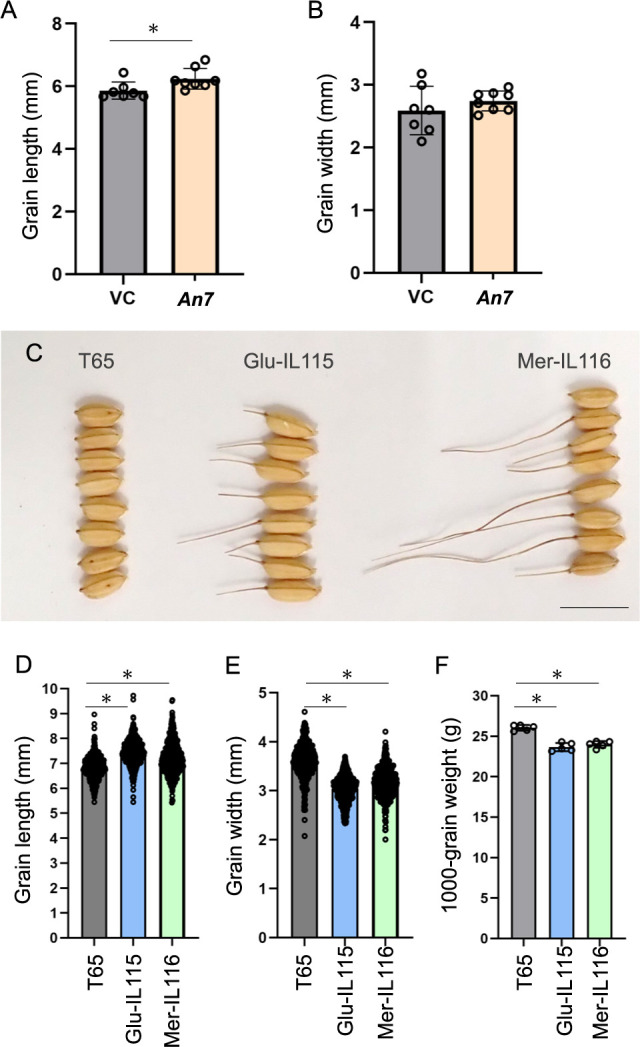
Grain phenotype of *O. sativa* and *An7* functional CSSLs. (A, B) Grain length (A) and grain width (B) of Vector Control (VC) and *An7* complementation line. (C) Grain characteristics of T65, Glu-IL115 and Mer-IL116. Scale bar, 10 mm. (D–F) Grain length (D), grain width (E), and 1,000-grain weight (F) of T65, Glu-IL115 and Mer-IL116. Data for grain length and width were measured from 500 seeds, and 1,000-grain weight data were measured over five trials. Error bars represent mean ± SD. Asterisks indicate P < 0.05 by student’s t-test.

The *d2* mutant, which is deficient in the biosynthesis of active BR, castasterone, presents a short grain phenotype (Hong *et al.* 2003). To clarify whether *An7* effects on grain phenotype, we measured grain size of *An7* complementation line and vector control (VC). The result indicated that the grain length of *An7* complementation line was longer than VC (Fig. 8A), but the grain width was not significantly different between both lines (Fig. 8B). This result suggested that *An7* could produce much BR for awn elongation and contribute pleiotropically to grain length elongation. To clarify the effect of *An7* on grain yield, we examined the grain size and yield in T65, Glu-IL115 and Mer-IL116 (Fig. 8C) due to the less enough number of seeds for measuring 1,000-grain weight of *An7* complementation lines. The grain lengths of Glu-IL115 and Mer-IL116 were significantly longer than T65 (Fig. 8D), but the grain widths of Glu-IL115 and Mer-IL116 were significantly narrower than T65 (Fig. 8E). The 1,000-grain weight of two CSSL lines decreased compared with T65 (Fig. 8F).

## Discussion

Understanding the regulation of awn development in rice is crucial for agricultural improvement. Awn presence plays a key role in deterring feeding damage from wild boars, particularly in the mountainous regions of Japan (Kanbe *et al.* 2011). On the other hand, the absence of awns in cultivated varieties facilitates more efficient harvesting and processing. Previous studies have identified several key genes associated with awn development, including *An-1/RAE1*, *RAE2/GAD1*, *LABA1* and *RAE3*, which play a role in awn regulation in wild rice species such as *O. rufipogon* and *O. barthii*, which are ancestors of domesticated rice species (Bessho-Uehara *et al.* 2016, 2023, Furuta *et al.* 2015, Hua *et al.* 2015, Jin *et al.* 2016, Luo *et al.* 2013). Despite these discoveries, there remains a significant gap in the research on this topic. Specifically, the genetic basis for awn regulation in other wild rice species remains unexplored. To address this knowledge gap, we investigated unidentified genetic loci involved in awn regulation. We focused on a range of wild rice species with the AA genome group that could be crossed with *O. sativa* which have no awns. A previous study, which evaluate several CSSLs crossed with five wild rice species belonging to AA genome species and fixed its background to *O. sativa* indicated that at least four loci related to awn length have not been identified (Bessho-Uehara *et al.* 2021). Among these loci, we identified the *An7* gene associated with awn elongation on chromosome 5 in *O. glumaepatula* and *O. meridionalis*.

*An7* encodes a *CYP90D3* gene. Its homologue, *D2/OsCYP90D2*, has been reported as a brassinosteroid (BR) biosynthesis enzyme involved in catalyzing specific steps in the late BR biosynthesis pathway, including the conversion of 6-deoxoteasterone to 3-dehydro-6-deoxoteasterone and teasterone to 3-dehydroteasterone (Hong *et al.* 2003). Considering the phenotype of *d2* mutant, which displays a semi-dwarf phenotype and shorter grain length (Hong *et al.* 2003), *D2* contributes to BR biosynthesis in multiple organs and resulted in a specific morphological phenotype. The grain lengths of *An7* complementation lines and both CSSLs carrying functional *An7* are longer than T65 (*O. sativa*). This suggested that *An7* may function pleiotropically not only to promote awn elongation but also grain length. However, it is important to note that due to the presence of multiple genes, besides *An7*, located on the substituted region of chromosome 5 in each CSSL, these may influence the final grain shape and yield. Consequently, based on the measurement of 1,000-grain weight in CSSLs alone, we cannot conclude whether functional *An7* negatively impacts yield.

There are two possibilities that *An7* allele of *O. sativa* to lose its function for awn elongation. One is the SNPs in the coding sequence of *An7* causing loss-of function as indicated in Fig. 4, and the other is a change in expression pattern compared to *O. glumaepatula* and *O. meridionalis*. A previous study showed that *An7*, which is annotated as *CYP90D3*, is expressed in root specifically in *O. sativa* (Hong *et al.* 2003). In young panicles, *An7* alleles of *O. glumaepatula* and *O. meridionalis* were highly expressed whereas *An7* allele of *O. sativa* was not. Some structural differences in the promoter sequence of *An7* in *O. sativa* compared to *O. glumaepatula* and *O. meridionalis* have the potential to alter the expression pattern of *An7*. Moreover, higher expression level of *An7* in Mer-IL116 than that of Glu-IL115 was consistent with the awn length (Fig. 1D, 1H). Since the plant height of Glu-IL115 and Mer-IL116 were the same as T65 (*O. sativa*), the expression pattern of *An7* would be restricted at awn and lemma/palea.

Several studies have provided support that implicates BR biosynthesis in awn development across different species. In barley, for instance, mutations in BR biosynthesis and receptor genes result in shorter awns (Dockter *et al.* 2014). Similarly, *An-1/RAE1* gene in rice, which encodes a bHLH-type transcription factor regulating BR response, was reported to positively regulate awn elongation (Furuta *et al.* 2015, Luo *et al.* 2013). In green foxtail (*Setaria viridis*), the *bristleless1* (*Bsl1*) gene has been identified as being responsible for bristle formation, which is an altered branch in inflorescences and shaped like awn by regulating BR biosynthesis at boundary regions (Yang *et al.* 2018). Taken together, these studies suggest BR is a positive regulator for awn and bristle development. Future experiments, such as quantification of endogenous BR in *An7* complementation line, or BR treatment to the spikelets on awnless varieties would allow us to verify the relationship between BR and awn development in rice.

Deciphering the genetic interactions underlying complex traits like awn length is crucial for a comprehensive understanding of their molecular mechanisms. Over ten QTLs have been reported for awn development in previous research (Cai and Morishima 2002, Fawcett *et al.* 2013). In this complex landscape, our study has added value by identifying *An7* as a new regulator that works cooperatively with *RAE3*. Taken together, these findings suggest that *RAE3* plays a critical role in awn elongation, even in species such as *O. glumaepatula*.

## Author Contribution Statement

MM, RM, MA and KBU designed the study. MM, RM and SA performed all the experiments. YS, YY, HY and AY provide the materials and performed sequence analysis. MM, RM and KBU wrote the manuscript. All authors have read and approved the final manuscript.

## Figures and Tables

**Table 1.** Primers used in this study

Purpose	Primer name	Sequence	Physical position (bp)
*An7* rough mapping marker	RM6320F	CGCTACGGAAGGGTAATAATGC	471,659
	RM6320R	AGCGTGGGAAGAAGGATACACC	471,838
	RM592F	ACCTCACCCGAATTACTGTGATATGC	2,796,922
	RM592R	GTTGAATTGCACGCGACTCTGG	2,797,118
	RM3328F	ATTGTCGAAGCAGCAAGAAAGG	5,266,943
	RM3328R	GACAATTGGAGTGTTGGACATGG	5,267,025
	RM3419F	TGCTGCTATTCCTCAAGACAAACC	5,284,902
	RM3419R	CTTGGTGAAACAGTGCTCTCTGG	5,285,075
	RM18055F	AGATCTCCTCTCAGAGTCTACCG	5,877,862
	RM18055R	CACTGAGTATAATCCCTGCAACC	5,878,157
	RM18059F	CGGAGGGTATGATCCGAAGAGG	5,965,605
	RM18059R	GCTGTGGTTGCTGCTGTTGTAGC	5,965,757
	6.097F	TGTGATGCTCTCCTATGCTC	6,097,290
	6.097R	GTTACCGAAAAGCAGTGGAC	6,097,394
	6.139F	CGATTCAGTGACCAATTTGC	6,138,951
	6.139R	TATGCGGATCTCTTCTCCTT	6,139,132
	6.172F	ACGTATAGCCGCTACTTTGG	6,172,945
	6.172R2	CCGGTTTTCGTCTATAACAT	6,173,074
	6.213F	GACTCGTGATGAACGAACTC	6,213,247
	6.213R	GTGCGCTATATATCTGCAAC	6,213,352
	6.223F	CAAGCTTGCAAGTAATTACG	6,222,952
	6.223R	CATTTTAATCAATTCCCTTTTG	6,223,012
	6.24F	GTAAGAATGTTGGTATCCAAC	6,246,212
	6.24R	GATTAAACCTATGTCCATGC	6,246,314
	6.324F	GGATCCACGCGCCTGGA	6,323,632
	6.324R	GACCCAACCAAGCAGAGAC	6,323,692
	RM7293F	CCTAGGGGATCCAAGATGTC	7,526,972
	RM7293R	GCACGGATCTACATACATGC	7,526,829
	RM5844F	AACGTGGCATCCATGTTAGTACC	9,149,506
	RM5844R	AGCTAGGAGCCATTGTCGAAGG	9,149,696
	RM5140F	GGCACTCGTATTTCTCAACTTCTCC	13,541,662
	RM5140R	GGGTGTATCAGGAGTACAGGTTGC	13,541,917
	RM18382F	GGAATTAAATGTGCGGGAATGC	14,824,645
	RM18382R	TGTAAGTACAAATCCGGCACCTATGG	14,824,842
	RM18550F	CCGAGATTGCAAGAATGAAGACAACC	18,080,743
	RM18550R	GCTGGCCATCGAGATGTTCG	18,080,963
	RM18551F	CGTGGCGAATAAATAGGCGAGAGG	18,081,391
	RM18551R	TGACCCTCTCCTGCCACCTACG	18,081,491
*An7* fine mapping marker *O. glumaepatula*	6.254_4_Hinf1_F	CAATGTGTCGGCAATGTCGC	6,254,284
	6.254_4_Hinf1_R	CAACCCGACCCATATTAGCC	6,254,536
	6.258_3_Msp1_F	AGTATGGGCCGCACATTAGG	6,258,155
	6.258_3_Msp1_R	TGCAAAATCAAGCAGGCAACA	6,258,313
	6.264F	GACTGGTTCTAATTGCGAGC	6,264,178
	6.264R	CCCTTAGCGCTGCATCAAAT	6,264,275
	6.267F	GGGCTTTTGCTTTAAAGAAG	6,267,114
	6.267R	GATAGAAACAGCTACAATGG	6,267,227
	6.291F	GAAAGCACGCAGCATATATAC	6,291,470
	6.291R	GCTCAAATAGCTAGTATTCCTAGTTC	6,291,548
	6.306F	CTTGCCACTGGTCAGACTAG	6,205,606
	6.306R	GTGAATGTAGGTTGTATGTGTTAAAG	6,305,670
*An7* fine mapping marker *O. meridionalis*	RM18058F	CGGTCCTCCACCTCAAGTCC	5,873,620
	RM18058R	CCCAGGGTAATGCAAGTGTAGC	5,873,639
	6.264F	GACTGGTTCTAATTGCGAGC	6,264,178
	6.264R	CCCTTAGCGCTGCATCAAAT	6,264,275
	RM18080F	GCGAAACTCATCGACTGATCG	6,323,956
	RM18080R	GGTGATCACATCATTGTCCATCC	6,323,979
	RM18076F	ACATGTCCCTGCCCGTAAACC	6,332,999
	RM18076R	GTCCACTCCAGCTCCTGCTTCC	6,333,162
	RM18107F	CGTATGGACTTGCCTTGAGTCG	6,827,017
	RM18107R	TCCAATCTGCCAAGCTTTACACC	6,827,311
*An7* candidate cloning	LOC_Os05g11120_Glu-IL115_F	TACTGTCGGCTGACCATGTTTTCTT	
	LOC_Os05g11120_Glu-IL115_R	ACCCTTGTTTGGGACTTTAGGGACT	
	LOC_Os05g11130_Glu-IL115_F	GCACCTTACCGATGTTCACCATCTACAT	
	LOC_Os05g11130_Glu-IL115_R	TGTCCGCCTTGCTATTCTGTACACATTT	
	LOC_Os05g11140_Glu-IL115_F	ACAGTCTAGACCCGGGATGGACGACGTCGACAGCG	
	LOC_Os05g11140_Glu-IL115_R	TGGCTGCAGGTCGACGTCATGACACCGTTCTTCCA	
	LOC_Os05g11160_Glu-IL115_F	ACACTATCGATCCACATTTGTTTTGAA	
	LOC_Os05g11160_Glu-IL115_R	TATGTCTTATCATCAACCCCATAACCA	
*An7* sequence primer	GLUM06720ORF seq R7.2-	TGCTCAGTACCATTGCAGTG	
	GLUM06720ORF seq R7-	AGTATGAGTACCATTTTCGTC	
	GLUM06720ORF seq F6-	GTTTGTGGCTTGGTGATGTAG	
	GLUM06720ORF seq R6-	TTACTACATCGATCCGTTTC	
	GLUM06720ORF seq F5-	AGTTGGTGCTTACTCTATCC	
	GLUM06720ORF seq R5-	CTCCATTTCTTTTACGTTGTC	
	GLUM06720ORF seq F4-	CTGTTAGCAAAACAACACTAC	
	GLUM06720ORF seq R4-	TCCAGCTTTTGGAAGAGAAG	
	GLUM06720ORF seq F3-	CTTTGTTCACTATTGGTTGAC	
	GLUM06720ORF seq R3-	GACACTTATAAACGGACAAACG	
	GLUM06720ORF seq F2-	GAATATGAGGAGTAGCTTGC	
	GLUM06720ORF seq R2-	CATCCTCTTCTTAGCCTAGATG	
	GLUM06720ORF seq F1-	TACCCACATGACAACCTCTC	
	GLUM06720ORF seq R1-	GTGCATGGGTCAACAGAATAG	
	GLUM06720ORF seq R1	ATTAGCTTAAGGAATCAGGACTTGGTC	
	GLUM06720ORF seq F2	GATATTCCAAGATTCACCCTACCGTATG	
*An7* qRT primer	An7qRT_F	AGACGCTGAGTTCATCTCC	
	An7qRT_R	TCCCCCATCAGCTCCATC	
	UBQ_F	GAGCCTCTGTTCGTCAAGTA	
	UBQ_R	ACTCGATGGTCCATTAAACC	
*RAE3* CRISPR gRNA	RAE3_CRISPR1_gRNA1	TGGAACCCATGGCGGCATTTCGG	
	RAE3_CRISPR1_gRNA2	CACCACGGCGCGGTCGACGCCGG	
	RAE3_CRISPR1_gRNA3	GGGATGCTTCGAGGCCGCCAAGG	
	RAE3_CRISPR2_gRNA1	TGGAACCCATGGCGGCATTTCGG	
	RAE3_CRISPR2_gRNA2	TGGGTTCCACGTCGAGTGCGTGG	
	RAE3_CRISPR2_gRNA3	CTCGAGTGCGCGGTGTGCCTCGG	
